# Methods of Isolation of Active Substances from Garlic (*Allium sativum* L.) and Its Impact on the Composition and Biological Properties of Garlic Extracts

**DOI:** 10.3390/antiox11071345

**Published:** 2022-07-09

**Authors:** Monika Bar, Urszula E. Binduga, Konrad A. Szychowski

**Affiliations:** 1 Department of Biotechnology and Cell Biology, Medical College, University of Information Technology and Management in Rzeszow, Sucharskiego 2, 35-225 Rzeszow, Poland; mbar@wsiz.edu.pl; 2 Department of Lifestyle Disorders and Regenerative Medicine, Medical College, University of Information Technology and Management in Rzeszow, Sucharskiego 2, 35-225 Rzeszow, Poland; ubinduga@wsiz.edu.pl

**Keywords:** garlic, bioactive compounds, solvent, extraction method, biological properties, bioavailability

## Abstract

Garlic (*Allium sativum* L.) is widely used in the human diet and in scientific research due to its biological properties. Various factors, e.g., temperature, pressure, extraction method, type of solvent, size, and territorial origin of garlic, affect the amount and type of bioactive compounds obtained from garlic extracts. In turn, the content of bioactive compounds correlates with the biological activity of the extracts. Therefore, the aim of this review was to summarize the current state of knowledge of the methods and effectiveness of isolation of active substances from garlic and their impact on the garlic extract composition and, consequently, biological properties. According to the literature, extracts obtained using water as a solvent are mainly responsible for antimicrobial properties, which is related to, inter alia, the high content of allicin. The use of alcohols, such as methanol or ethanol, is associated with the outstanding antioxidant power of extracts resulting from the presence of phenolic compounds. In turn, due to the presence of diallyl disulfide and disulfide trisulfide, garlic oil has anticancer potential. Acetone is the most effective organic solvent; however, it is not suitable for immediate consumption.

## 1. Introduction

Garlic (*Allium sativum* L.), i.e., a bulbous plant from the *Lillaceae* family, is native to central Asia but at present grows in many countries around the world [1]. Garlic is widely used due to its seasoning and flavor qualities as well as health-enhancing properties [2]. Moreover, it is used in folk medicine as a remedy for the treatment of bacterial and viral diseases [3,4,5]. 

There are many consumable forms of garlic on the market, i.e., fresh garlic, garlic extract, garlic oil, dehydrated oil macerate, temperature aging garlic bulbs, and garlic powder [6,7]. However, garlic is used for therapeutic purposes in tablet, oil, and powder forms [6]. Currently, highly efficient methods are being sought to obtain active ingredients from various vegetables or fruits [8]. It has been shown in the literature that, in the case of both fruit and vegetables, the extraction method and the type of solvent affect the content of bioactive ingredients and biological properties [9,10,11,12]. Bioactive compounds isolated from plants, including garlic, are especially used in the pharmaceutical, food, and cosmetic industries [13]. Factors that influence the isolation and extraction efficiency include weighing, volume measurement, mixing, dilution, heating, cooling, fractionation, purification, and preservation [13]. Each solvent used in the extraction of active compounds is characterized by different polarities, which may be important in terms of the solubility of bioactive compounds and, consequently, the extraction efficiency [14,15]. Moreover, the volume ratio of individual solvents (in extractions with solvent mixtures) is also important and may correlate with the amount of recovery of individual bioactive compounds [15]. For this reason, the choice of the extraction method should be based on the following factors: the nature of the plant material, the nature of isolated compounds, the impact of the extraction method on the degradation of compounds, and the development of undesirable microorganisms [13]. Moreover, the method of isolation of active ingredients should be safe for consumers and acceptable for human consumption, e.g., as a food additive.

**Figure 1:**
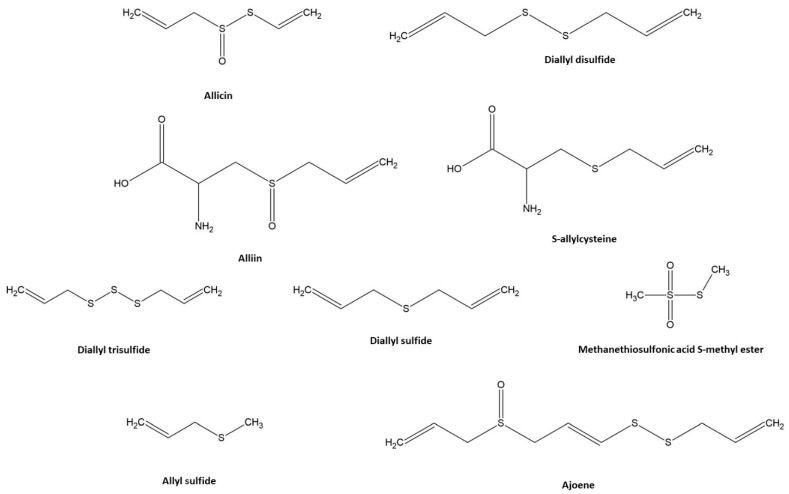
Main sulfur compounds identified in garlic: allicin (AC), alliin, S-allylcysteine (SAC), diallyl disulfide (DADS), diallyl trisulfide (DATS), diallyl sulfide (DAS), methanethiosulfonic acid S-methyl ester (MMTS), allyl sulfide (AS), and ajoene.

Garlic consists of 60–65% of water, 28–30% of carbohydrates, ~2.3% of organosulfur compounds, 2–6% of proteins, ~1.2% of amino acids, and ~1.5% of fiber, fatty acids, phenols, and mineral trace elements [6,16,17]. However, the composition of different garlic varieties may vary significantly [18,19]. In addition, the composition of garlic bulbs is strongly influenced by the soil and broad-sense weather and climatic conditions [18,19]. Garlic owes its main healing properties to diverse bioactive compound groups, such as organic sulfides, saponins, phenolic compounds, and polysaccharides [20,21]. Moreover, the group of sulfur compounds identified in garlic has been shown to comprise a variety of active compounds, e.g., allicin (AC), alliin, S-allylcysteine (SAC), diallyl disulfide (DADS), diallyl trisulfide (DATS), diallyl sulfide (DAS), and ajoene [22] (Figure 1).

A substantial number of studies have shown that, thanks to its unique composition of bioactive constituents, garlic exhibits antibacterial, antifungal, immunomodulatory, antiinflammatory, antioxidant, anticancer, hepatoprotective, gastroprotective, cardiovascular protective, neuroprotective, renal protective, antidiabetic, anti-obesity, and anti-coagulant properties. It has also been used in such diseases as cholera or malaria and to reduce LDL cholesterol [1,22,23,24,25]. Moreover, according to the World Health Organization (WHO), the American Cancer Society (ACS), the American Institute of Cancer Research (AICR), and the National Cancer Institute (NCI), garlic consumption may reduce the risk of cancer [26].

Therefore, the aim of the present publication is to collect and summarize the current knowledge of the methods of extraction, with particular emphasis on the influence of the solvent used during the isolation of active substances from garlic (*Allium sativum* L.) and their impact on the composition and biological activity of extracts.

This review, in an innovative way, summarizes not only the biological properties of garlic extracts but mainly focuses on the significant influence of the optimization of extraction parameters. Different parameters influence the quantity, quality, type, and biological properties of the extracted bioactive compounds from the plant material. The review compares traditional extraction methods with modern ones. Knowledge concerning the efficiency of extraction, bioavailability, and stability of bioactive compounds is crucial to overcoming the difficulties in the delivery of bioactive compounds in final therapeutic applications.

## 2. Methods of Isolation of Active Compounds

Bioactive compounds are obtained with the use of different extraction methods and types of solvents, which affect the extraction yield and biological properties of extracts [15]. The disadvantages of traditional extraction methods include high temperature during the process, large amounts of solvents used, the toxicity of some solvents, long processing times, and low selectivity [27]. The advantage of the new extraction methods is the reduction in the amount of solvents, shorter required process time and higher yield and quality of the obtained extract [28]. Water, methanol, ethanol, and acetone are most often used for the extraction of bioactive compounds from plants (Table 1). 

The selection of the method and type of solvent is important and depends on the types of plant material and isolated compounds [15]. The use of water has numerous advantages, such as the positive environmental impact (water is non-flammable and non-toxic to humans and the environment, provides the possibility of clean processing and prevents pollution), selective extraction of bioactive compounds (depending on the type of solvent, it allows avoiding extraction of unwanted components) [38,39,40]. Additional advantages include the use of simple equipment, no risks, simplification of process steps, and the possibility of application in the food and pharmaceutical industries [39,40]. In addition to water, acetone, ethyl acetate, hexane, heptane, dichloromethane, methanol, ethanol, tetrahydrofuran, acetonitrile, dimethylformamide, toluene, and dimethylsulfoxide are commonly used organic solvents [41]. Despite the high levels of extraction of certain substances, the main disadvantage of these solvents is the danger to health through ingestion and inhalation, as well as irritation in contact with the skin and possible damage to the central nervous system (CNS) and other parts of the body through regular environmental exposure to these compounds [41]. 

**Figure 2:**
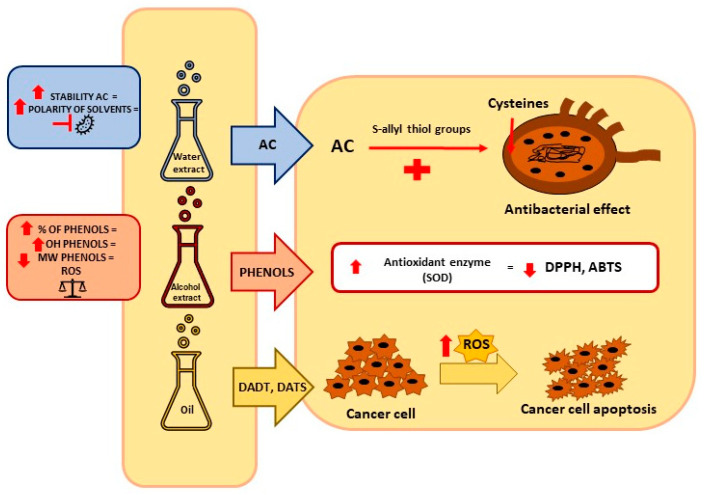
Influence of the solvent used in extraction on the type of bioactive compounds and biological properties. Abbreviations: ABTS—2,2′-Azino-bis(3-ethylbenzthiazoline-6-sulfonic acid; AC—allicin; DADS—diallyl disulfide; DADT—disulfide trisulfide; DPPH—2,2-diphenyl-1-picrylhydrazyl; MW phenols—molecular weight of phenols; OH phenols—hydroxyl group of phenols; ROS—reactive oxygen species; SOD—superoxide dismutase.

The choice of other parameters of the extraction method is important as well. Traditional methods of obtaining bioactive compounds from plants are Soxhlet extraction, distillation, infusion, and cold pressing. On the other hand, modern, green, environmentally friendly extraction methods include ultrasound-assisted extraction (UAE), high pressure-assisted extraction (HPAE), microwave-assisted extraction (MAE), enzyme assisted extraction (EAE), supercritical fluid extraction (SFE), pulse electrified field extraction (PEF), pressurized liquid assisted extraction (PLE), and surfactant-assisted extraction (SAE). The current use of the traditional extraction methods results from the possibility of using simple equipment and, depending on the type of plant and the extraction purpose, from the better stability of the extracted compounds. Often, traditional methods such as Soxhlet extraction or distillation in combination with modern ultrasonic or microwave technologies prove to be the most effective. Despite the many advantages of modern extraction methods, the optimization of time, temperature, and solvent parameters in the adaptation to the plant type is important for each method and contributes to quality and efficiency [42]. Recent technological advances and the development of devices ensure greater extraction efficiency [43]. UAE, MAE, PLE, and SFE can change the content of bioactive compounds, including AC [44]. Loghmanifar et al. [45] showed that, of the extraction methods used, such as immersion, cooking, and ultrasound with the use of water/ethanol solvents, ultrasonic extraction was the most effective. The main advantages of this method are its speed, efficiency of the process, and the use of a lower temperature during extraction, which is important in the case of heat-sensitive bioactive compounds [28]. The most commonly used methods for extracting garlic oil are steam hydrodistillation and hot solvent extraction. Water temperature, distillation time, and particle size are factors that influence the efficiency of hydrodistillation extraction [43]. The temperature, the analytical degree of the solvents, the maceration time, and the drying method are important factors in the extraction of garlic oils with organic solvents [43]. According to the results obtained by Chen et al. [46], parameters ensuring the best quality of garlic oil extraction with the use of solvents are as follows: temperature 45 °C, time 42 min, solvent to raw material ratio 4:1, number of extractions 4 [46]. In addition to the influence of the extraction type on the content of organic compounds, the method of preparation of biological material is also important. According to the literature, alliin and γ-glutamyl-cysteine (γGCS) derivatives are the main bioactive compounds obtained from fresh garlic bulbs. Steam distilled oils are rich in sulfide compounds, ground powder is a source of alliin and DADS, and macerates contain mainly sulfide compounds, dithin, and (E-Z)-ajoene compounds. In turn, soaked, cut, and aged garlic extract using ethanol as a solvent is a source of bioactive compounds such as SAC and S-allyl-mercaptocysteine (SAMC) [47,48]. The summary of the influence of solvents on the isolation of bioactive compounds and the biological properties of extracts is presented in Figure 2.

### 2.1. Aqueous Solutions

The main advantage of aqueous solutions is the possibility of direct consumption or administration to animals or cells in vitro without additional processing [49]. The evaluation of bioactive ingredients in water extracts from fresh garlic varieties NSPBL-70, Labud, and Bosut showed the highest amount of AC, ranging between 42.74 and 50.79 μg/mL, compared to other bioactive compounds present in the extract. The content of methanethiosulfonic acid S-methyl ester (MMTS), allyl sulfide (AS), and DADS was in the range of 0.09–0.33 μg/mL, 1.91–4.72 μg/mL, and 0.01–0.03 μg/mL, respectively. However, the value differed depending on the variety of garlic [9]. Similarly, water extracts obtained from lyophilized garlic powder exhibited the highest content of AC = 12.35 ng/mg, while the levels of MMTS, AS, and DADS were on average 1.42, 1.31, and 0.16 ng/mg, respectively [9]. In turn, a comparative analysis of ultrasound-assisted extraction (60 Hz, room temperature) with the use of water, ethanol, acetone, and mixtures of the solvents carried out by Cavalcanti et al. [15] proved the effectiveness of the water-based extraction method. The use of water resulted in the highest recovery of thiosulfinates (TS) at a level of 6.42 µmol/g dry weight (dw), while the amount of total phenolic compounds (TPC) expressed as gallic acid equivalent (GAE) was 3.82 mg/g dw [15]. A high extraction yield in a process with the use of water as a solvent (26.50%) from garlic shell (GH) was also demonstrated by Kallel et al. [50]. The yield was higher than in the extraction procedure based on the use of other solvents: ethanol, methanol, ethanol-water, and methanol-water. Nevertheless, the water extract was characterized by the lowest total content of phenols and flavonoids, i.e., 2.97 mg and 0.045 mg expressed as quercetin equivalent per gram (QE/g) of dry GH, respectively [50]. This was most likely related to the better solubility of the tested bioactive compounds (e.g., polyphenols) in solvents of lower polarity than water and the nonpolar nature of GH [50]. As demonstrated by Meriga et al. [51], the content of bioactive compounds in hexane, chloroform, ethyl acetate, methanol, and water extracts varied [51]. The presence of steroids, alkaloids, flavonoids, carbohydrates, tannins, and glycosides has been demonstrated in the aqueous garlic extract. Moreover, flavonoids, alkaloids, carbohydrates, as well as tannins and glycosides, were present only in the water and methanol extracts [51]. As shown by Kaur et al. [52], aqueous extracts also contained phthalic acid derivatives, acid esters, compounds containing phenyl groups, and steroids, which induced antimicrobial response of aqueous garlic extract against *Bacillus anthracis* [52]. In turn, the study described by Szychowski et al. [20] showed that water extracts from 9 varieties of garlic from Poland, Spain, China, Portugal, Burma, Thailand, and Uzbekistan were rich in a protein mixture and polyphenols [20]. Szychowski et al. [20] showed that the quantitative differences between these compounds depended on the variety of garlic. The highest content of peptides was obtained from Chinese and Spanish garlic extracts, i.e., 6.12 mg/mL and 4.87 mg/mL, respectively. Similarly, the Chinese and Spanish garlic extracts were also the richest in protein content, i.e., 2.80 mg/mL and 2.81 mg/mL, respectively. Moreover, as demonstrated by the biochemical analysis of phenolic compounds, the Chinese and Spanish garlic extracts had the highest amount of polyphenols, i.e., 394.10 μg/g and 365.52 μg/g of raw garlic, respectively. Syringic and p-hydroxybenzoic acid derivatives were found in the water extract in the greatest amount. Gallic acid, p-coumaric acid, k(+)-catechin, and epicatechin were the other phenolic compounds occurring in extracts from some garlic varieties [20]. As shown by Loghmanifar et al. [45], the highest content of phenolic compounds in aqueous ultrasonic extract (40 kHz for 15 min) was 0.311 mg GAE/g, which was higher than their content in 50% ethanol ultrasonic extract (40 kHz for 15 min). In addition, aqueous extract obtained in a shaking incubator (40 °C for 72 h) contained a high phenolic amount of 0.295 mg GAE/g. On the other hand, a lower amount of phenols (0.191 mg GAE/g) was determined in the aqueous extract subjected to thermal treatment in an oven (35 °C for 24 h), and the lowest level (0.112 GAE/g) was recorded for cooked aqueous extract. Finally, the influence of both methods and solvents on the phenol content in the garlic extract was confirmed [28]. Due to the high potential of the ultrasonic water extract, further analysis of the bioactive compounds was carried out and revealed the highest contents of DADS (34.87%), dipropyl trisulfide (25.88%), pyrogallol (13.38%), and methyl propyl trisulfide (11.36%) [28]. In turn, Johnson et al. [53] determined the levels of carbohydrate (66.8%), oil (2.6%), moisture (14.5%), total ash (1.3%), and protein (14.8%) in garlic [53]. The phytochemical analysis of the water extract showed the presence of bioactive ingredients, i.e., steroids, protein (high content), tannins, terpenoids, saponins (moderate content), reducing sugar, and phenols (low content) with the presence of phenols and flavonoids equal to 0.285 mg/mL and 28.74 mg QUE/mL, respectively, which are responsible for antimicrobial and antioxidant properties [53]. An effect of the use of various solvents (ethanol, diethyl ether, acetone, hexane, water) on the solubility of the phytochemicals and, consequently, on the antimicrobial properties was demonstrated as well [54]. The aqueous extract contained carbohydrates, total protein, saponins, and tannins but did not show the presence of alkaloids and steroids; hence, ethanol extracts are the richest sources of a variety of secondary plant substances (carbohydrates, total protein, saponins and tannins, alkaloids, and steroids) among all analyzed garlic bulb extracts [54].

### 2.2. Alcohol Solutions

The content of bioactive compounds in ethanol extracts (96% ethanol) was determined by Bajac et al. [9], who used 3 varieties of garlic (NSPBL-70, Labud, and Bosut) and showed an amount of AC ranging from 4.39 to 4.56 μg/mL. The levels of other components, i.e., MMTS, AS, and DADS, were in the range of 0.45–0.67 μg/mL, 0.21–0.70 μg/mL, and 0.03–0.04 μg/mL, respectively, depending on the type of garlic [9]. In turn, lyophilized garlic ethanol extracts were distinguished by the MMTS content of 3.33 ng/mg compared to AC = 1.05 ng/mg, AS = 0.99 ng/mg, and DADS = 0.57 ng/mg [9]. Caiñzares et al. [55] showed a notable AC level of 7068 ppm in the ethanol extract (96%, *v*/*v*) [55]. Moreover, similar to acetone, ethanol ensured high efficiency of extraction of thiosulfinates (such as AC) compared to water/hexane-based extraction. Storage did not increase the yield, which may be related to the ability to recover the solute only from the outer layer surrounding vegetable particles [55]. In turn, in the study conducted by Mamun et al. [56], polyphenols, terpenoids, steroids (absence in the petroleum ether extract), saponins, tannins (absence in the acetone extract), flavonoids, alkaloids, and glycosides (absence in the acetone extract and petroleum ether extract) were detected in both ethanolic and methanolic extracts [56]. The following bioactive compounds were isolated from the ethanol extract (99.80%): phenols 24.81 mg expressed as gallic acid GAE/g (dry extract), 22.51 mg flavonoids expressed as CAE catechins/g (dry extract), flavonols 12.92 mg expressed as quercetin QUE/g (dry extract), and proanthocyanidins 5.13 mg CAE/g (dry extract). The following compounds were detected in the methanol extract: phenols—29.72 mg GAE/g (dry extract), flavonoids—20.18 mg CAE/g (dry extract), flavonols—11.92 mg QUE/g (dry extract), and proanthocyanidins—5.17 mg CAE/g (dry extract) [56]. Higher efficiency of TPC and TS recovery was found for ultrasound-assisted extraction with ethanol (99.80%) than for acetone and ethanol-acetone extraction (50%:50%, *v*/*v*). The presence of TPC and TS in the ethanol extract was 0.84 GAE/g dw and 1.40 µmol/g dw, respectively. Higher TPC and TS recovery was recorded in the other tested solvents (water) and solvent mixtures (water, ethanol, acetone) [15]. In turn, Kallel et al. [50] showed a lower yield in ethanol and methanol extracts than that in the other studied garlic GH extracts, equal to 4.00% and 7.00%, respectively. The higher extraction efficiency of bioactive compounds from garlic was achieved with the use of the following solvents: 50%:50% water-ethanol (*v*/*v*)—20.00% and 50%:50% water-methanol (*v*/*v*)—7.33% [50]. This experiment showed that water improved the ethanol extraction yield through the increased polarity of the solvent. Other factors that affect the yield of extraction are high temperature and the solid-to liquid-ratio [50]. The methanol-water extract showed the highest recovery of phenols (25 mg GAE/g dry GH) and flavonoids (0.617 mg QE/g dry GH). Similarly, the total contents of phenols and flavonoids in the methanol extract were high, i.e., 22.83 mg GAE/g dry GH and 0.60 mg QE/g dry GH, respectively. In turn, the use of ethanol as a solvent showed the presence of phenols and flavonoids at a level of 11.80 mg GAE/g dry GH and 0.49 mg QE/g dry GH, respectively. In the case of the water-ethanol solvent, the phenol content was 13 mg GAE/g dry GH and the content of flavonoids was 0.51 mg QE/g dry GH [50]. Further analysis of the composition was then performed for the 50%:50% water-methanol extract (*v*/*v*), the 50%:50% water-ethanol extract (*v*/*v*), and the methanol extract [50]. The presence of phenolic acids and hydroxycinnamic acids such as ferulic acid, gallic acid, hydroxybenzoic acid, caffeic acid, p-coumaric acid, di-ferulic acid, chlorogenic acid, caffeic acid O-glucoside, coumaroylquinic acid, coumaric acid O-glucoside, and caffeoylputrescine was demonstrated for methanol-water extract. A similar composition was found for the ethanol-water extract except for the addition of cinnamic acid and synapic acid [50]. A high yield of extracts is achieved by the addition of water to acetone, ethanol, and methanol solvents, thus increasing polarity, which facilitates the extraction of bioactive compounds such as phenols [50]. On the other hand, the analysis of the composition of the methanol extract showed the presence of phenolic acids and hydroxycinnamic acids, as in the case of the 50%:50% methanol-water extract (*v*/*v*), with the exception of the absence of caffeic acid O-glucoside and coumaric acid O-glucoside [50]. In turn, Meriga et al. [51] proved the presence of steroids, triterpenes, flavonoids, alkaloids, saponins, tannins, and glycosides in extracts obtained using methanol as a solvent [51]. Bin et al. [54] reported the presence of carbohydrates, alkaloids, total protein, saponins, tannins, and steroids in the ethanol extract [54]. In contrast, the ethanolic ultrasonic extract contained a lower amount of phenols than in water-based isolation, i.e., 0.269 mg GAE/g, while the amount of phenols in the oven ethanolic extraction was 0.216 mg GAE/g [28] (Table 2).

### 2.3. Other Organic Solvents

Unfortunately, organic solvents are dangerous to humans and are not suitable for direct consumption due to their toxicity, which is influenced by their concentration, time of exposure, frequency, and nature. In addition, the solvent must be removed, which may result in degradation or loss of active substances [41]. In the study conducted by Cañizares et al. [55], an AC level of 3663 ppm was detected in the acetone extract (99.50%, *v*/*v*) [55]. The yield of the acetone extraction of bioactive compounds was significantly higher (38.18%) than in the other tested extracts (2–6%) (ethanol + Soxhlet method; ethanol + Stirred tank extraction (ET); hexane + Soxhlet method; hexane + ET; water with solvent elimination; water without solvent elimination). This is related to the degradation of the cell wall and, consequently, the recovery of the solute from garlic cells through acetone extraction. As a result, the water content decreased, and the porosity increased during storage. Therefore, the efficiency of acetone extraction increased with storage for up to 9 months [55]. As in the case of using water as a solvent, the efficiency of the extraction process with hexane (99.50% *v*/*v*) was low [55]. Mamun et al. [56] also investigated the presence of bioactive compounds in acetone (99.50%), chloroform, and petroleum ether extracts in comparison to aqueous, ethanol, and methanolic extracts [56]. The authors found polyphenols, terpenoids, steroids, saponins, tannins, flavonoids, alkaloids, and glycosides in the acetone extract. The presence of the same compounds was identified in the chloroform extract and petroleum ether extract, except for the absence of tannins and glycosides in the chloroform extract and the absence of steroids and glycosides in the petroleum ether extract [56]. Moreover, further quantitative analysis of the acetone extract revealed characteristic contents of phenolic compounds—110.76 mg GAE/g (dry extract), flavonoids—43.32 mg CAE/g (dry extract), flavonols—15.31 mg QUE/g (dry extract), and proanthocyanidins—8.54 mg CAE/g (dry extract). The chloroform extract showed the presence of 41.19 mg GAE/g of phenols, 25.70 mg CAE/g of flavonoids, 9.00 mg QUE/g of flavonols, and 3.62 mg CAE/g of proanthocyanidins. In turn, the petroleum ether extract contained 25.65 mg GAE/g of phenolic compounds, 14.39 mg CAE/g of flavonoids, 5.81 mg QUE/g of flavonols, and 3.00 mg CAE/g of proanthocyanidins [56]. By analogy with the ethanol extract analyzed by Cavalcanti et al. [15], the use of acetone (99.50%) in ultrasonically assisted extraction resulted in lower content of TPC and TS equal to 0.35 mg GAE/g dw and 1.43 µmol/g dw, respectively, compared to the other tested extracts (water, ethanol) and mixtures of extracts [15]. The acetone extract showed only a higher level of TPC and TS compared to the lowest content of these compounds in the 50%/50% ethanol-acetone extract [15]. In analyses of hexane, chloroform, and ethyl acetate extracts, saponins were found in the hexane extract and ethyl acetate extract, but steroids and triterpenes were detected in the chloroform extract, showing the different affinity of these compounds for certain solvents. The presence or absence of individual compounds in the extracts is associated with their biological activity [51]. However, other studies have shown quite a different extraction profile, in which in acetone (carbohydrates, total protein, steroids, flavonoids), diethyl ether (carbohydrates, total protein, tannins, steroids), and hexane extracts (carbohydrates, alkaloids, total protein, flavonoids) were analyzed [54].

Additionally, Cavalcanti et al. [15] analyzed solvent mixtures using ultrasonic extraction and the single-sided axial design (SAD) method [15]. The authors showed the highest recovery of TPC (5.84 mg GAE/g dw) in a water-ethanol-acetone mixture (66.6%:16.7%: 16.7%, *v*/*v*/*v*) compared to the lower level obtained through water extraction, ethanol extraction, acetone extraction, and in other solvent mixtures. The TS content in the water-ethanol-acetone mixture (66.6%:16.7%:16.7%, *v*/*v*/*v*) extraction was 3.69 µmol/g dw, and a higher amount of TS was found only in the water extract [15]. The water-ethanol and water-acetone mixtures (50%:50%, *v*/*v*) were also rich in bioactive compounds. The contents of TPC and TS were 3.62 mg GAE/g dw and 2.78 µmol/g dw in the water-ethanol extraction, respectively, and 4.28 mg GAE/g dw and 3.41 µmol/g dw in the water-acetone variants. In turn, the acetone-ethanol mixture (50%:50%, *v*/*v*) yielded the lowest amount of TPC (0.26 mg GAE/g dw) and TS (0.33 µmol/g dw) [15]. 

### 2.4. Garlic Essential Oil Quality Depending on the Extraction Method

Garlic oils are obtained by steam distillation of garlic cloves using n-hexane or petroleum ether, among other methods [57]. The amount of oil in garlic cloves is in the range of 0.20–0.50%, and DADS and DATS are the bioactive compounds in the oil. Due to its pungent odor, garlic oil capsules contain mainly vegetable oils and lower content of garlic oil [57]. As reported by Dehariya et al., garlic oil is rich in DADS (48.42%), allyl-methyl trisulfide (7.27%), trisulfide, di-2-propenyl (3.46%), and DAS (7.64%) [58]. Rafe et al. studied the effect of the extraction procedure and solvent type on the physicochemical properties of garlic oil that can facilitate the encapsulation process [23]. The yield of garlic oil extraction expressed as the volume of oil after evaporation of the solvent was dependent on the extraction method used, with the highest result of 7.00% for supercritical fluid extraction (SCF). The oil obtained with the SCF method exhibited high viscosity, which facilitated encapsulation. Extraction with solvents and steam distillation indicated a yield level of 6.00% and 5.50%, respectively. The specific gravity of garlic oils, which is important for commercial feasibility, was also checked, but the method showed no effect on its value (0.894 g/cm^3^). The higher efficiency achieved by SCF compared to solvent extraction and steam distillation may be related to better transport properties, such as diffusivity, mass transfer coefficient, and penetration capacity [23].

### 2.5. Other Factors

Parameters such as temperature, pressure, and the size of the garlic used in the extraction are important for the extraction process [27,59]. Aqueous extraction in a stirred tank with a solvent recovery step resulted in a loss of antimicrobial activity with a maximum holding temperature of the extract of 95 °C and an overall extraction temperature of 21 °C. For comparison, aqueous extraction was performed without solvent recovery at a lower extraction temperature of 23 °C, and the extract exhibited antimicrobial activity against *Helicobacter pylori* [55]. As reported by the authors, the negative effect of the temperature close to boiling is the result of the thermal degradation of compounds responsible for inhibition of the growth of bacteria [55]. The negative effect of temperature on the content of bioactive compounds and enzymes involved in their production was also reported by Loghmanifar et al. [45], who showed the lowest AC content of 0.009% in boiled water extract, which confirms the negative effect of heat [28]. In addition, cooking softens the cell wall and facilitates the release of carotenoids into the water during extraction, which results in their lower content in tissues [28]. In turn, the highest AC content, equal to 0.086%, was detected in the ultrasonic aqueous extract [28]. On the other hand, Pedraza-Chaverrı et al. [60] compared the following extracts: cooked garlic clove extract, microwave-treated garlic clove extract, pickled garlic extract, heated garlic powder extract, and heated raw garlic extract [60]. They demonstrated the thermal stability of the bioactive compounds in the aqueous garlic extract involved in scavenging superoxide anion (O_2_•^−^), hydrogen peroxide (H_2_O_2_), and hydroxyl radicals (OH•). No effect of alliinase on the scavenging of the tested radicals was shown [61]. However, compared to unheated raw garlic or unheated garlic powder, the O_2_•-scavenging capacity of the microwave-treated garlic clove extract and the heated raw garlic extract was lower. In turn, a lower antioxidant potential in relation to H_2_O_2_ scavenging was demonstrated for the heated garlic powder extract and the pickled garlic extract [61]. The extraction method has an impact on the acquisition of components as well. It has been proven that the extraction efficiency in a tank with an agitator is more effective than that of the Soxhlet extraction due to the higher yield, lower operating temperatures (room temperature), and easy operation [55]. The effective mixing speed was 175 rpm, and the extraction time was 2 h. The selected mixing speed provides suitable efficiency, whereas a higher speed could impede solute recovery. On the other hand, extending the extraction time to 3 h does not significantly increase the efficiency but increases the costs of the extraction process [55]. The increase in the extraction efficiency is influenced by the particle size of garlic, which increases with the decrease in size. This is associated with the larger specific surface area and, as a result, easy removal of the contents [55]. Dehariya et al. [58] analyzed the effect of 2, 3, and 4 h garlic ethanol extraction with the Soxhlet method at temperatures of 50, 60, and 70 °C on the efficiency and antioxidant properties [58]. The highest yield, i.e., 16.55%, was obtained after 4 h of extraction at 50 °C. In turn, the highest antioxidant properties (12.018 mM of tocopherol per mL of oil) were achieved by extraction for 2 h at 70 °C. As suggested by the authors, it may have been a result of the higher activity of sulfur compounds and phenols or better extraction at 70 °C [58]. Another study compared the extraction of garlic using ethanol as a solvent with supercritical carbon dioxide (SC-CO_2_) extraction [59]. Despite the higher yield of the ethanol extraction (5.50%) than the SC-CO_2_ extraction, where the yield ranged from 0.65 to 1.00%, ethanol turned out to be a less selective solvent for the valuable components of the extract [59]. Moreover, in the case of SC-CO_2_, it was found that a temperature in the range of 35–60 °C at a pressure of 300 bar had little effect on the extraction rate and yield. On the other hand, with the increase in the pressure in the range of 150–400 bar at a constant temperature of 50 °C, an increase in the extraction efficiency was observed. However, the efficiency of extraction by a further increase in the temperature led to thermal damage and/or co-extraction of unwanted compounds resulting in degradation and/or dilution of the desired components [59]. In addition, the comparison of raw materials in the form of fresh crushed garlic and dehulled or dried garlic showed a greater similarity to commercial preparations of fresh garlic. This may be explained by the thermal or oxidative degradation of precursors and products during hot air drying resulting in the loss of valuable bioactive compounds [59]. Among the methods of allicin extraction, i.e., solvent extraction, ultrasonic-assisted extraction (UAE), pressurized liquid extraction (PLE), supercritical CO_2_ extraction (SCCO_2_), and subcritical water extraction (SWE), a great potential of the SWE method has been shown by researchers, considering the disadvantages of the other methods, i.e., the use of an organic solvent, the long extraction time, and the application of two types of processes (enzymatic process and extraction process). Moreover, the advantage of the SWE method is the ability to regulate the temperature in the subcritical phase, which in turn facilitates the modification of the polar character of water and, consequently, the possibility of efficient extraction of compounds with medium and low polarity [62].

## 3. Biological Properties of Garlic Extracts

### 3.1. Antibacterial Properties

**Figure 3:**
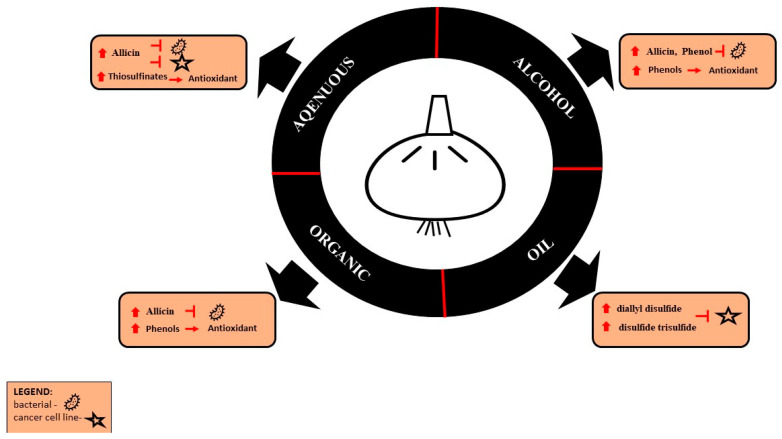
Mechanism of biological activity of the main bioactive compounds contained in extracts and oils of garlic (*Allium sativum* L.).

The high antimicrobial effectiveness of garlic is related to the content of such compounds as allicin, ajoenes, and allyl sulfides. It has been well described that AC inhibits the growth of both Gram-positive and Gram-negative bacteria and reduces the formation of bacterial biofilm [63]. AC is one of the main bioactive compounds in garlic water extracts [57]. When garlic is chopped and/or crushed, AC is produced through the activation of the alliinase enzyme, which acts on alliin [64]. According to the literature, extracts obtained with the use of water as a solvent are characterized by a particularly high antimicrobial potential, which is correlated with the high AC content [9,65]. The antimicrobial activity of alcohol extracts and organic extracts has been demonstrated as well [55]. In addition, despite its low polarity, AC is more stable in higher polarity solvents such as water at 0.1 MPa than in lower polarity organic solvents [28,66]. Table 3 summarizes the biological properties of *Allium sativum* L. in both in vivo and in vitro tests. The mechanism of biological activity of the main bioactive compounds contained in extracts and oils of garlic (*Allium sativum* L.) is shown in Figure 3.

#### 3.1.1. Antibacterial Activity of Aqueous Extracts

The antimicrobial activity of aqueous garlic extracts against *Escherichia coli* expressed as the minimum inhibitory concentration (MIC) was estimated at 56.82 to 227.27 μL/mL, while the minimum bactericidal concentration (MBC) ranged from 227.27 to 454.54 μL/mL depending on the garlic variety. In turn, MIC and MBC of aqueous extracts against *Staphylococcus aureus* ranged from 56.82 to about 120.00 μL/mL and from about 120.00 to 227.27 μL/mL, respectively, also depending on the garlic variety [9]. Moreover, the researchers compared the antimicrobial activity of the analyzed water and ethanol extracts and showed a higher efficiency of the water extracts, which was correlated with higher AC content, with a 10-fold difference between the extracts [9]. The antimicrobial properties of aqueous extracts of garlic were investigated by Wallock-Richards et al. [65], who also highlighted the correlation of antimicrobial activities with the presence of AC in the extract [65]. The MIC interval ranged from 0.50 to 3.00% (*v*/*v*) for 38 *Burkholderia cepacia* complex isolates. Moreover, a further mechanism of action of AC with a recombinant form of thiol-dependent peroxyredoxin (BCP) indicated that both pure AC and AC from garlic aqueous extract modified the cysteine residue of BCP. As suggested by the authors, AC may act as an electrophilic reagent targeting protein thiols [65]. On the other hand, Cañizares et al. observed that water extract was less effective than ethanol and acetone extracts in terms of the antimicrobial properties against *Helicobacter pylori* [55]. Similarly, despite the high yield of aqueous extract with antibacterial activity, Kallel et al. [50] demonstrated lower antibacterial efficacy than that of extracts obtained with the use of other solvents. This may be correlated with the biochemical composition of the extract, i.e., the phenolic content and high polarity of water, which hinders the extraction of nonpolar compounds from GH [50]. In turn, Olukunle and Adenola [117] showed higher effectiveness of aqueous extract of *Allium sativum* compared to methanol and ethanol extracts against two clinical strains of *Salmonella typhi* [117], with the efficiency of the aqueous extract (21.83%) and the growth inhibition zone at the concentration of 800 mg/mL equal to 7.00 mm and 10 mm for *S. typhi* I and K isolates, respectively. In turn, the MIC for *S. typhi* I and K isolates was 150.00 mg/mL, and the MBC was 200.00 mg/mL [117]. The mechanism of the antimicrobial action of polyphenols is mainly based on the inhibition of microbial extracellular enzymes, effects on metabolism, and deprivation of substrates that are necessary for microbial growth [118]. Among the tested extracts from 14 plants, the water extract of garlic most effectively inhibited the development of *B. anthracis*, responsible for the development of gastrointestinal anthrax, which underlines the importance of these extracts [52]. The antimicrobial activity of the aqueous extract against *B. anthracis* was confirmed in the agar well diffusion test (AWDA), while the evaluation of the biocidal activity showed a decrease in the number of viable colony-forming cells/mL (CFU/mL) by 6 logs from 6 to 12 h in liquid cultures exposed to the aqueous extract. Moreover, the evaluation of the extract stability proved its thermostability at 50 °C for 12 h with the antimicrobial effect > 80.00%. Additionally, the potential interaction of the aqueous extract with antibiotics currently used in the treatment of anthrax was tested, and no antagonistic activity was found. Finally, it was proved that mainly derivatives of phthalic acid, acid esters, compounds containing phenyl groups, and steroids are responsible for the antimicrobial activity of water garlic extracts [52]. Moreover, similar to methanol extract, the water extract showed high antimicrobial efficacy, which was higher than that of the hexane extract. Chloroform and ethyl acetate had negligible activity. The use of water as a solvent most effectively inhibited the growth of *Bacillus subtilis*, *Staphylococcus aureus*, *Escherichia coli*, *Klebsiella pneumoniae*, and *Candida albicans*. The highest sensitivity was exhibited by *B. subtilis* with an inhibition zone of 20 mm and a minimum inhibitory concentration of 100 µg/mL [51]. Johnson et al. [53] also demonstrated the antimicrobial efficacy of an aqueous extract of garlic with a growth inhibition zone of 25.6 mm for *S. aureus* and 28.1 mm for *P. aeruginosa*. In this study, the MIC and MBC values were 80 mg/mL and 104 mg/mL, respectively, for *S. aureus* and 40 mg/mL and 88 mg/mL, respectively, for *P. aeruginosa*. These data indicate the greater sensitivity of *P. aeruginosa* to the antimicrobial properties of the aqueous extract [53]. In addition, Durairaj et al. [119] proved the antimicrobial efficacy of aqueous garlic extract against 15 g-positive bacteria and 2 g-negative bacteria. The tested aqueous extract inhibited the growth of *Bacillus subtilis* most strongly but had the least potent inhibitory activity against *Proteus* sp. The MIC value was in the range of 6–11 mg/mL for Gram-positive bacteria and 7–21 mg/mL for Gram-negative bacteria [119]. Moreover, the influence of temperature and time on the antimicrobial properties of the aqueous extract was investigated, and maintenance of the biological properties was demonstrated at room temperature for up to 7 days and at −20 °C for 90 days [119]. In addition, aqueous ultrasonic garlic extracts have shown an antimicrobial potential to inhibit the growth of *E. coli*, *Staphylococcus aureus* sub. *aureus*, *Streptococcus mutans*, and *Poryphyromonas gingivalis* strains [69].

#### 3.1.2. Antibacterial Activity of Alcoholic Extracts

Similar to water extracts, ethanol extracts also have strong antimicrobial activity. Bajac et al. [9] assessed the antibacterial properties of ethanol extracts with MIC and MBC of 113.64 μL/mL and 227.27 μL/mL, respectively, for Gram-negative *E. coli*. For Gram-positive *S. aureus*, the type of garlic used was significant, with MIC ranging from 113.64 to 227.27 μL/mL and MBC in the range from 227.27 μL/mL to 454.54 μL/mL [9]. Additionally, as in the case of water extracts, a possible influence of the presence of AC on antimicrobial properties was found [9]. In turn, Cañizares et al. [55] proved the effectiveness of ethanol extract in inhibiting the growth of *H. pylori*, *E. coli*, and *Staphylococcus epidermidis*. The antimicrobial properties are most likely correlated with the AC content [55]. Comparative analysis with antibiotics proved that garlic extract was more effective than nalidixic acid and metronidazole, and its effectiveness was comparable to that of ciprofloxacin and erythromycin. Considering the commercial concentrations of erythromycin (15 µg), metronidazole (10 µg), ciprofloxacin (10 µg), and nalidixic acid (30 µg), as well as the quantity of the extracts used, the garlic extracts were shown to have only 3- or 4-fold lower antimicrobial activity [55]. Antibacterial activity against Gram-positive bacteria *B. subtilis*, *S. aureus*, and *Bacillus thuringiensis* and Gram-negative *P. aeruginosa* and *Klebsiella pneumoniae* was exhibited by methanol extract [50]. Gram-positive bacteria were more sensitive to the action of the extract than Gram-negative bacteria, which is related to the difference in the composition of their cell envelope [50]. In turn, Olukunle and Adenola (2019) examined the antibacterial activity against *S. typhi* and showed lower antimicrobial efficiency and potency of ethanol and methanol extracts in comparison with aqueous garlic extract [117]. Bin et al. [54] demonstrated the antimicrobial activity of ethanol, diethyl ether, acetone, hexane, and water extracts against drug-resistant bacterial strains isolated from periodontal and dental caries samples (*Lactobacillus acidophilus, Streptococcus sanguis, S. salivarius, S. mutans*, and *Staphylococcus aureus*) [54]. The ability to produce biofilm and the production of extracellular polysaccharides (EPS) by bacteria contributes to the currently increasing resistance to antibiotics. As shown by the researchers, the ethanol extract was the most effective, especially against *S. mutans* with MIC of 20 mg/mL and MBC of 70 mg/mL, as well as *S. aureus* with MIC of 35 mg/mL and MBC of 60 mg/mL [54]. *Allium sativum* ethanol extract in a concentration of 20% showed a strong antibacterial effect against *Ralstonia solanacearum* [120]. In recent studies with formocresol as a positive control and ethanol as a negative control, garlic ethanol extracts in concentrations of 100%, 50%, and 25% showed antimicrobial activity against *S. mutans* and *Lactobacillus acidophilus*. The latter was a more sensitive strain. Formocresol at a concentration of 1:5 showed excellent antimicrobial activity, and no such activity was observed with the use of ethanol at 24 and 48 h intervals. As reported by Vyas et al. [121], *Allium savitum* ethanol extracts may be a replacement for the currently used formocresol in the treatment of pulpotomy [121]. In turn, methanol extract and water-methanol (50%:50%) extract induced strong inhibition of bacterial growth, which was related to the high content of phenols in the extracts such as caffeic acid, p-coumaric acid, ferulic acid, and di-ferulic acid [50]. In addition to the antibacterial activity of the aqueous-methanol extract (50%:50%) against the Gram-positive strains tested, this extract applied at a concentration of 10 mg/mL was effective against Gram-negative strains of *P. aeruginosa* and *K. pneumoniae* [50]. Lower antimicrobial effectiveness and lower content of phenolic compounds were found for the ethanol extract. In addition, the lower phenol content in the ethanol-water extract (50%:50%) was correlated with the lower antimicrobial potential [50]. Moreover, Meriga et al. [51] demonstrated antimicrobial activity of methanol extracts against *B. subtilis*, *E. coli*, and *K. pneumoniae*. As in the case of water extraction, *B. subtilis* was the most sensitive organism, with an inhibition zone of 16 mm and a MIC value of 100 (µg/mL). The methanol extract exhibited no antimicrobial efficacy only against *S. aureus* and *C. albicans* [51].

The antimicrobial properties of alcohol extracts are mainly associated with the content of AC and polyphenols [9,50]. However, despite the AC content in alcoholic extracts (e.g., ethanol extracts), usually aqueous extracts show stronger antimicrobial activity, most likely due to the presence of other compounds in the extract and their synergistic or additive effect [63].

#### 3.1.3. Antibacterial Activity of Other Types of Extracts

High activity inhibiting the growth of *H. pylori* was exhibited by acetone and ethanol extracts, whereas hexane and water extracts were characterized by low efficiency [55]. The high activity is most likely related to the AC and allyl-methyl and methyl-allyl thiosulfinate content in the extracts [55,122]. Similar to ethanol, acetone extracts were estimated to have 3 to 4 times lower activity against *H. pylori* compared to the commercial concentrations of the tested antibiotics [55]. Moreover, both acetone and ethanol extracts were effective and inhibited the growth of *E. coli* and *S. epidermidis* in addition to *H. pylori* [55]. In turn, Meriga et al. [51] reported no antimicrobial activity of hexane, chloroform, and ethyl acetate garlic extracts against *B. subtilis, S. aureus, E. coli, K. pneumoniae*, and *C. albicans* [51].

### 3.2. Antioxidant Properties

Bioactive compounds such as polyphenols play a protective role against oxidative stress-related diseases, in addition to traditional antioxidants, e.g., butylated hydroxyanisole (BHA), butylhydroxytoluene (BHT), and ascorbyl palmitate (PA) [123]. As reported in the literature, ethanol and methanol garlic extracts usually have strong antioxidant properties, which result from the high content of phenols [50]. Aqueous extracts and extracts obtained using organic compounds as solvents also exhibit antioxidant activity [51,56]. The antioxidant efficiency in scavenging free radicals, e.g., DPPH•, depends on the quantitative content of phenols and tocopherols in the tested extracts [56]. An increase in the concentration of phenols is accompanied by an increase in the antioxidant power [28,124]. An increase in the number of hydroxyl groups and in the concentration of phenols in extracts results in an increase in the potential of hydrogen donors for free radicals. Antioxidant activity depends on the position of phenol hydroxyl groups, and phenolic compounds with lower molecular weights exhibit their greater bioavailability [28]. The study of Ahmad et al. [125] assessed the effect of methods, i.e., maceration, percolation, ultrasonic-assisted, Soxhlet and Soxtec extraction (STE), and accelerated solvent extraction (ASE), on the extract yield and phenolic content of *Allium sativum* L. In addition to the above, the effect of the type of solvents such as n-hexane, dichloromethane and methanol, and temperature (60, 80, and 100 °C) on the following factors such as extraction yield was evaluated, phenolic content and antioxidant activity (DPPH, and ABTS). Both STE and ASE methods using methanol as solvent at 100 °C showed high yield and recovery of the extract equal to 1221.10 mg/5 g (24.42%) and 91.50 mg/1 g (9.15%), respectively [125].

In addition to their antimicrobial properties, water extracts have antioxidant power. This is most likely related to the high content of bioactive compounds such as TS [15]. The total antioxidant capacity (TAC) and oxygen radical absorbance capacity (ORAC) were 200.31 mg expressed as ascorbic acid equivalent (AAE)/g dw and 882.47 μmol expressed as Trolox equivalent (TE)/g dw, respectively, and were higher than in the case of the acetone-water mixture (1/4: 3/4, *v*/*v*) [15]. Further antioxidant analysis also showed the ability to scavenge the DPPH• radical with an IC_50_ of 3.40 mg/mL [15]. In contrast, Kallel et al. [50] showed a lower antioxidant efficiency of water extracts compared to methanol, ethanol, and 50%:50% methanol-water, which was related to the low phenol content [50]. As in the case of antimicrobial properties, the higher efficiency of lower polarity solvents than water is most likely a result of the nonpolar nature of GH [50]. In addition to the strong antioxidant activity exhibited by methanol extract, Meriga et al. [51] demonstrated high antioxidant efficiency of aqueous garlic bulb extract, which was confirmed by the high DPPH• radical scavenging ability in the range of 80.00–90.00%. The strong reducing power of the aqueous extract, which amounted to 55.00–65.00%, was higher than that demonstrated for the methanol extract in relation to ascorbic acid [51]. The garlic varieties originating from different geographic areas used for the extraction had an effect on the antioxidant power [20]. The highest DPPH• and ABTS radical scavenging capacity was exhibited by the Chinese extract with values of 4.63 μg/mL and 0.43 μg/mL, respectively. Moreover, this extract contained the highest content of peptides, proteins, and polyphenols. The greatest power of chelation of Cu^2+^ ions was exhibited by the extracts from the Polish (16.58 μg/mL) and Chinese garlic (14.90 μg/mL) [20]. In turn, as reported by Loghmanifar et al. [45], the ultrasonic water extract and the shaking water extract showed the highest reduction in the DPPH• radical amounting to 50.00% at 500 ppm; analogously, the highest IC_50_ antioxidant activity of 4.376 µg/mL was determined for the aqueous ultrasonic extract compared to the other tested extracts (ultrasonic ethanol, oven aqueous, oven ethanol). In turn, the lowest antioxidant power IC_50_ of 8.540 mg/mL was demonstrated for the boiled aqueous extract, which corresponds to the lowest phenol content in this extract. The phenol content in extracts has an influence on the antioxidant effectiveness [28]. Another study also showed the potential of garlic aqueous extract in the concentration range from 3 to 40 mg/mL to reduce the DPPH•^+^ radical from 4.47% to 92.44%. The IC_50_ for the aqueous extract was 25.30 mg/mL [53]. The phenolic constituents found in plants, including flavonoids, phenolic acids, and phenolic diterpenes, are mainly responsible for the antioxidant activity [53,126]. This is related to the redox properties of phenols, which result in neutralization of free radicals, quenching of singlet and triplet oxygen, and breakdown of peroxides [53]. 

On the other hand, as the concentration of ethanol and methanol extracts (µg/mL) increased, the antioxidant activity and iron-reducing antioxidant power increased [56]. In order to analyze the antioxidant potential of garlic extracts obtained using various solvents, the following tests were performed: DPPH•, ABTS, hydroxyl radical scavenging (HO•), and lipid peroxidation (LPI) tests. The antioxidant activity of ethanol extract with IC_50_ (50% inhibitory concentration) was 7.80 µg/mL (DPPH•), 13.60 µg/mL (ABTS), 16.70 µg/mL (HO), and 21.70 µg/mL (LPI), respectively. In the case of methanol extract, it was equal to 7.20 µg/mL (DPPH•), 15.90 µg/mL (ABTS), 16.30 µg/mL (HO), and 22.50 µg/mL, respectively (LPI) [56]. Similarly, Kallel et al. [50] proved the antioxidant effectiveness of extracts obtained using methanol as a solvent [50]. This was evidenced by the high DPPH• radical scavenging capacity with an IC_50_ of 0.64 mg/mL and the hydroxyl radical scavenging capacity of 90.04% at 3 mg/mL. Moreover, a strong reduction potential of Fe^3+^ to Fe^2+^ was found [50]. The antioxidant efficacy of ethanolic and methanolic garlic extracts was reported by Mamun et al. [56] and Kallel et al. [50] (methanol extract), showing the dependence of the antioxidant activity on the phenol content in the tested extracts [50,56]. Likewise, Meriga et al. [51] demonstrated the antioxidant potential of methanol extract with DPPH• radical scavenging capacity ranging from 80% to 90% and 40% to 50% reducing power compared to ascorbionic acid [51]. The antioxidant potential of 50%:50% methanol-water extract (*v*/*v*) related to the high total recovery of phenolic compounds was also demonstrated by Kallela et al. [50]. The 50%:50% (*v*/*v*) methanol-water extract was characterized by IC_50_ for DPPH• of 0.26 mg/mL, high potential of reduction in free hydroxyl radicals, and reducing power equal to 1.69 at 10 mg/mL. In contrast, the 50%:50% ethanol-water (*v*/*v*) extract exhibited lower antioxidant activity, reflected in the IC_50_ value for DPPH•, and free radical scavenging potential, which were 1.26 mg/mL and 0.344 at 10 mg/mL, respectively. The reducing power of this extract was lower as well [50]. Similarly, the antioxidant potential of methanol extract (98.00%) from garlic reflected as the ability to scavenge the DPPH• radical was estimated by Sultana et al. [127] at IC_50_ of 89.25 µg/mL, compared to that of ascorbionic acid [127].

As reported by Mamun et al., an increase in the concentration of extracts with the use of organic solvents results in an increase in the antioxidant potential and ferric reducing antioxidant power [56]. This is particularly evident in the case of acetone extract, which showed higher antioxidant activity than the commonly used BHT antioxidant, with IC_50_ of 5.10 µg/mL for DPPH•, 11.30 µg/mL for ABTS, 15.70 µg/mL for HO^.^, and 19.50 µg/mL for LPI. The effectiveness of chloroform and petroleum ether extracts was lower [56]. Most likely, this is the result of a quantitative difference in the phytochemical composition (phenols). The IC_50_ value in the DPPH•, ABTS, HO, and LPI tests was 18.50 µg/mL, 47.60 µg/mL, 38.40 µg/mL, and 77.60 µg/mL, respectively, for the chloroform extract and 19.90 µg/mL, 27.90 µg/mL, 50.10 µg/mL, and 91.10 µg/mL, respectively, for the petroleum ether extract [56]. Moreover, the DPPH• radical scavenging ability of the acetone-water extract (25%:75%), which was higher than in water extraction, also proves the high biological activity with IC_50_ at the level of 2.88 mg/mL. The antioxidant power was evidenced by the TAC and ORAC values of 171.30 mg AAE/g dw and 794.63 µmol TE/g dw, respectively [15]. 

A positive correlation was found between the type of extraction, the yield of the extract, and the content of phenolic compounds, which was not the case between the antioxidant activity and the type of extraction for the STE method [125]. In the case of the ASE method, there was a positive correlation between the solvent and the extraction efficiency, phenols, and antioxidant activity, and no correlation was found between the extraction efficiency and DPPH activity. In addition, for both STE and ASE, low IC_50_ values (μg/mL) were found at 1.09 and 1.18 for DPPH, 2.11 and 5.32 for ABTS, and 4.35 and 7.88 for phenazine methosulfate-nicotinamide adenine dinucleotide [125].

As demonstrated by Sani et al., methanolic extracts of garlic bulbs containing phenolic compounds at the level of 4.273 mg GAE/g dw increased the activity of the antioxidant superoxide dismutase (SOD) enzyme by 60% and reduced the level of total cholesterol (TC) by 34% in Wistar rats with Alloxan-induced diabetes [128]. In turn, Nasr observed the therapeutic effects of ripe garlic extract on hepatotoxicity induced by the use of the anticancer drug cisplatin (CP). Garlic extracts decreased the level of malondialdehyde (MDA) and increased the levels of the following antioxidant enzymes: catalase (CAT), SOD, and reduced glutathione (GSH), which proves the antioxidant properties of garlic in in vitro conditions [129].

### 3.3. Anticancer Properties

Garlic oils have an anticancer potential, which correlates with the content of bioactive compounds such as DADS and DATS. The antitumor effect has also been demonstrated for water extracts [130]. The antitumor mechanism is mainly related to the activation of reactive oxygen species (ROS) production, which in turn leads to apoptosis of cells treated with oil or garlic extract [37,131]. Both polyphenols and flavonoids, including those isolated from plants, have antioxidant potential (e.g., they scavenge endogenous ROS) and pro-oxidative potential, which can be used in cancer therapy [132,133]. Lee et al. (2013) used reversed-phase high-performance liquid chromatography (TLC), thin-layer chromatography (TLC), mass spectrometry (MS), nuclear magnetic resonance (NMR), chemical synthesis, and cell viability (MTT) assay, and isolated AC with antitumor potential from aqueous garlic extract [30]. The mechanism of the anticancer activity of bioactive sulfur garlic compounds, such as DAS, DADS, DATS, alliin, SAC, SAMC, and AC, involves alteration of mitochondrial permeability, inhibition of angiogenesis, enhancement of antioxidant and proapoptotic properties, and regulation of cell proliferation [1]. The correlation of the antioxidant potential with the antitumor activity of aqueous garlic extract was also demonstrated by Avci et al. [134] in two mouse cell lines, 32D (normal cells) and 32Dp210 (chronic myelocytic leukemia cells) [134]. There was no change in the activity of the xanthine oxidase (XO) enzyme and antioxidant enzymes (SOD, glutathione peroxidase (GSH-Px), CAT), while there was an increase in malondialdehyde (MDA) in the 32D cells. In turn, an increase in XO, antioxidant power, and MDA was demonstrated in the 32Dp210 cells. In addition, the researchers demonstrated antiproliferative and apoptotic activity in the 32D and 32Dp210 cells. The 0.4% (*w*/*v*) concentration of the aqueous extract proved to be the most effective, with a 2-fold higher apoptosis rate in the 32Dp210 versus 32D cells [134]. Szychowski et al. described that water extracts of the Polish garlic cultivar “Harnaś” and the Spanish variety “Morado” exhibited strong anticancer properties in the human squamous cancer cell line (SCC-15) measured by ROS production, lactate dehydrogenase (LDH) release, caspase-3 activity, and neutral red uptake methods [130].

Jasamai et al. [31] analyzed the antitumor effect of garlic methanol extract (99.50%) on the viability and apoptosis of the U-937, Jurkat Clone E6-1, and K-562 leukemia lines. The researchers determined IC_50_ values for U-937 (105 µg/mL), Jurkat clone E6-1 (489 µg/mL), and K-562 (455 µg/mL). The Jurkat clone E6-1 cell line appeared to be the most sensitive to methanol extract, showing a 38.37% increase in apoptosis compared to the control. In the other leukemia lines tested, apoptosis was estimated at 17.93% for the U-937 cells and 16.37% for the K-562 cells. Moreover, 6.87% necrosis was demonstrated after treatment of the U-937 cells with the methanol extract [31].

Aquilano et al. [131] proved the antitumor efficacy of DADS in the neuronal SH-SY5Y and NSC34 cell lines. This mechanism was based on cell apoptosis and an increase in the level of ROS and nitric oxide [131]. In turn, Choi et al. [37] showed the effect of the presence of DATS in garlic oils on the apoptosis in leukemic cells and the increase in ROS levels. The mechanism of apoptosis in U937 cells was the result of a decrease in the level of Bcl-2, XIAP, and cIAP-1 proteins, Bid cleavage, caspase activation, and a breakdown of the mitochondrial membrane potential [37]. In addition, Zeng et al. demonstrated the beneficial effects of garlic oil consumption during radiotherapy and chemotherapy. Although garlic oil did not increase tumor inhibition by CTX/radiation, it inhibited the decrease in peripheral white blood cells, DNA content, and bone marrow micronucleus ratio [135]. The anticancer effects of garlic extracts on individual cell lines are presented in Table 4.

### 3.4. Other Biological Activities of Garlic Extracts

The biological properties of garlic described below emphasize its importance, paying particular attention to the dose responsible for the beneficial or toxic effect. A number of factors (choice of solvents, extraction methods, and temperature) exert an impact on the amount and type of compounds extracted from garlic and, consequently, on the biological properties and bioactivity of compounds contained in garlic extracts.

#### 3.4.1. Antiviral, Antifungal, Antiparasitic, and Insecticidal Properties

Garlic extracts also have antiviral properties, i.e., they were found to inhibit infectious bronchitis virus (IBV) cultivated in a chicken embryo, as demonstrated by Mohajer Shojai et al. [146]. Garlic, and especially its organosulfur compounds (OSC), stop viral infections by inhibiting viral penetration into host cells, viral RNA polymerase, reverse transcriptase, DNA synthesis, and transcription of immediate-early gene 1 (IEG1). Another mechanism of antiviral properties described by Rouf et al. [147] is the downregulation of extracellular signal-regulated kinase (ERK)/mitogen-activated protein kinase (MAPK) signaling [147]. Recent reports also indicate a strong antiviral effect of garlic against SARS-CoV-2 infection [106,107,108]. Bioactive compounds, such as allyl disulfide and allyl trisulfide present in garlic oil with a content of 51.3%, showed the strongest activity against SARS-CoV2. According to the docking results, researchers demonstrated a synergistic mechanism of 17 organosulfur compounds present in garlic oil by inhibiting host receptor angiotensin-converting enzyme 2 (ACE2) protein and protease (PDB6LU7) proteins [107]. In turn, as demonstrated by Pandey et al. [106], bioactive compounds derived from *Allium sativum*, and in particular alliin, are a potential inhibitor of the main protease COVID-19 due to their ability to bind to the 6LU7 protein [106].

As reported by Bakhshi et al. [148], water garlic extract has antifungal potential and can be used in the treatment of denture stomatitis (DS) caused by *Candida* yeasts [148]. This extract does not show side effects. In comparison, nystatin, i.e., a commonly used antibiotic in the treatment of DS, may cause an allergic reaction, adrenal insufficiency, liver necrosis, and drug interactions, and nystatin tablets have a bitter taste [148]. Garlic extracts also have the potential to treat onychomycosis through their antifungal activity against such yeasts as *Meyerozyma guilliermondii* and *Rhodotorula mucilaginosa*, in which changes related to cell death were found at *Allium sativum* extract MIC of 120 mg/mL [25]. As demonstrated by Pârvu et al. [25], *Allium sativum* extract has comparable antioxidant efficacy to that of the bioactive compound AC and the drug diclofenac in the treatment of mycosis [25].

Meriga et al. [51] observed high insecticidal efficacy of water extract of garlic bulbs against *Spodoptera litura*, which was correlated with the composition of the extract. The larval mortality was estimated at 64.00%, 55.00%, and 42.00% at concentrations of 1000 ppm, 500 ppm, and 250 ppm, respectively [51]. In turn, similar to aqueous extracts, methanol extracts also showed high insecticidal effectiveness, compared to the lower insecticidal effectiveness for extracts obtained using hexane, chloroform, and ethyl acetate. The larval mortality increased with the increasing concentration of the extracts and was 81%, 72%, and 66%, respectively, using methanol as a solvent at 1000 ppm, 500 ppm, and 250 ppm. The insecticidal activity was correlated with the high content of phytochemicals and the interaction between garlic compounds [51].

#### 3.4.2. Antiinflammatory Activity and Treatment of Cardiovascular Diseases

The antioxidant properties of garlic extracts can be used in the treatment of inflammation caused by turpentine to reduce the oxidative state in serum, decrease the levels of MDA and nitric oxide (NO), and increase the level of thiols [25]. The correlation between oxidative stress and inflammation may be related to ROS-induced activation of transcription factors and pro-inflammatory genes [149]. The antiinflammatory mechanism of garlic extracts was also described by Arreol et al. [150]. It involves the engagement of bioactive compounds present in garlic in the inhibition of the transcription of cytokine genes, e.g., tumor necrosis factor-α (TNF-α), interleukin-1beta (IL-1β), IL-6, monocyte chemoattractant protein-1 (MCP-1), and IL-12 [47]. Keiss et al. [93] demonstrated the antiinflammatory properties of garlic powder extract, which at a concentration of 100 mg/L reduced the level of lipopolysaccharide (LPS)-induced pro-inflammatory cytokines such as interleukin (IL)-1β from 15.7 to 6.2 μg/L and tumor necrosis factor (TNF)-α from 8.8 to 3.9 μg/L in human whole blood [93]. As shown by these researchers, the garlic bioactive compound DADS contained in the extract in the concentration range of 1–100 μmol/L reduced (IL)-1β and TNF-α levels [93]. This was correlated with the presence of sulfur, as HEK293 kidney cells exposed to blood supernatant treated with sulfur-unfertilized garlic (100 mg/L) exhibited a 25% reduction in the activity of NF-κB, whereas a 41% decrease in this activity was shown for blood supernatants treated with an extract from sulfur-fertilized garlic (100 mg/L) [93]. Another study showed the antiatherosclerotic potential of both the ethanol extract and the bioactive compound SAC itself, i.e., reduction in the cytotoxic effect caused by oxidized LDL (Ox-LDL) on endothelial cells (EC) [151]. Treatment of the cells with the extract or SAC resulted in the prevention of glutathione (GSH) depletion and reduction in peroxides. Moreover, SAC itself inhibited the activity of NF-κB, which was induced by H_2_O_2_ or TNF-a [151]. 

Garlic extracts also have the potential for the treatment of cardiovascular diseases through their antihypertensive activity. Different studies examined the effect of fermented garlic extract on the circulatory system by focusing on anti-pressure activity [152,153]. Mun Park et al. [152] demonstrated the sGC-cGMP-PKG pathway-mediated antihypertensive potential of nitrites contained in fermented garlic extract, which are converted into NO in the body [152]. Mun Park et al. [152] proved the beneficial properties of fermented garlic extract (0.97 mg of nitrite/mL/day) in the reduction in monocrotaline-induced hypertension in rats (50 mg/kg). The mechanism of action was based on the reduction in the inflammatory response via the NO-sGC-PKG pathway [153]. In turn, Ushijim et al. [154] showed inessential anti-pressure effectiveness of a sulfur compound present in mature garlic extract, i.e., S-1-propenylcysteine at a dose of 6.50 mg/kg body weight, which significantly reduced systolic blood pressure in Wistar Kyoto rats with spontaneous hypertension [154]. A drop in blood pressure of about 10.00% was achieved 3 h after administration of the compound to the rats, and a return to the initial value was observed after 24 h. The authors did not observe significant changes in the heart rate. Further analysis of such sulfur compounds present in the extract as SAC and SAMC showed no therapeutic effect [154]. In turn, antihypertensive activity was demonstrated by Ried et al. [155] by administration of four tablets containing 960 mg of garlic extract and 2.40 mg of SAC per day to a group of 50 humans for a 12-week treatment period. The garlic extract turned out to be well tolerated by the patients, and a 10.20 mmHg decrease in systolic blood pressure was demonstrated in the hypertensive subjects (≥140 mmHg) after 12 weeks, compared to the control group [155].

#### 3.4.3. Neuroprotective Properties

The broad neuroprotective effect of SAC contained in garlic extract was demonstrated by Zarezadeh et al. [156] in lipopolysaccharide (LPS)-induced cognitive deficit. Administration of SAC (100 mg/kg/day) for 7 days resulted in improved memory of spatial recognition in the Y-maze, discrimination factor in the new object recognition task, and retention and recall in the passive-avoidance test in rats [156]. The mechanism of the positive action of SAC consisted of the reduction in oxidative stress by weakening the lipid peroxidation marker MDA and activation of SOD, CAT, and GSH in the rat hippocampus [156]. The other defense mechanisms of the nervous system include the reduction in neuritis, astrogliosis, and acetylcholinesterase [156]. In turn, the properties of the garlic extract described by Jing-FangLuo et al. [157] indicate its possible use in Alzheimer’s disease due to the reduction in cognitive impairment and neuropathology, for example, by reducing the level of beta amyloid (Aβ40 and Aβ42) [157].

#### 3.4.4. Other Health Properties of Garlic Extracts 

A hepatoprotective effect was demonstrated for garlic oil, and the bioactive component DADS contained therein. In a murine model, Yi-Syuan et al. [158] reported anti-obesity and antihyperlipidemic effects, evidenced by the reduced body weight and adipose tissue mass as well as serum biochemical parameters after treatment with both garlic essential oil and DADS, which has protective potential against the occurrence of nonalcoholic fatty liver disease (NAFLD) [158]. In addition, both the oil (50 and 100 mg/kg) and DADS (20 mg/kg) reduced the level of pro-inflammatory cytokines in the liver, which ultimately resulted in the inhibition of cytochrome P450 2E1 expression [158]. Despite all these advantages of garlic extract, special attention should be paid to the dose, which may be responsible for both therapeutic and toxic effects [159]. The garlic extract administered to rats (500 and 1000 mg/kg) by Siddique et al. [159] exerted a toxic effect, i.e., the occurrence of hemorrhages and nodular edema as well as an increase in body and liver weight [159].

## 4. Bioavailability of Garlic Extracts

Phadatare et al. [160] searched for a form of delivery of AC, which is highly unstable also at low temperatures. AC is generated through the interaction of the alliinase enzyme with the alliin precursor [160]. In addition, alliinase is destroyed by gastric juice. Intestinal fluid inhibits the release of AC from the powder in standard formulations by up to 40%, while intestinal epithelial cells degrade AC [160]. On the other hand, garlic oil is poorly bioavailable after oral administration [160]. In order to overcome these difficulties, the authors proposed and prepared buccal tablets containing freeze-dried garlic powder. The buccal tablets consisted of allicin-releasing garlic powder, methocel K4M, carbopol 974 P, mannitol, and magnesium stearate. The advantages of this form of AC were that freeze-drying inhibited the effect of powder alliinase due to the lack of water [160]. Satisfactory physicochemical properties of the tablets were achieved at the following parameter values: AC content 78.66 (µg/tablet), mucoadhesive strength 7.28 (gm), and 22.65% of absorbed moisture. In turn, the better bioavailability of garlic oil was improved by the use of self-emulsifying systems. Nanoemulsions were absorbed on colloidal silicon dioxide and then placed in hard gelatin capsules. Such nanoemulsions had a high absorption surface and, consequently, suitable bioavailability in oral administration. The capsules consisted of garlic oil, Cremophor EL, Transcutol P, and Aerosil 200 [160]. In addition to the relevance of the extraction method on the isolation of the bioactive compounds from garlic and their biological properties, it is necessary to pay attention to the method of delivery of the bioactive compounds from the extracts or whole garlic extracts and oils to living organisms in order to ensure the highest possible efficacy. The efficacy of the delivery method of crude garlic extract is important, as demonstrated by Li et al., [161], who showed an antitumor effect after intravenous administration of the extract at 100 mg for 21 days in mice, while oral administration showed no therapeutic against fatal ascites tested on the 180 (aggressive lethal mouse sarcoma) and EL4 (aggressive mouse lymphoma) cancer cell lines [161]. This may be associated with metabolic changes in epithelial cells after oral administration as well as the effects of digestive enzymes and the acidic environment of the stomach. A comparison of plain crude extract and extract heated at 100 °C for 10 min showed partial inactivation of some tumor lines after heating. Furthermore, in vitro anticancer activity studies on various cell lines confirmed the superior efficacy of garlic extracts compared to 21 fruit and vegetable extracts [161]. In addition, further research is carried out on the encapsulation and use of nanoparticles for garlic extracts or oils for potential use as preservatives and further possible use for drug delivery [162,163,164].

## 5. Conclusions

Depending on the solvent used for the extraction, the resulting extracts differ in the content of different active substances. This manuscript summarizes the extraction methods used to obtain bioactive substances from garlic (*Allium sativum* L.). The literature study showed that aqueous extracts are the most proper for allicin extraction, while methanol- and ethanol-based extraction processes are best for the isolation of polyphenols. The content of other active substances in the extract also depends on the used solvent. In water extracts, active substances can be ranked AC > AS > MMTS > DADS. This proportion is similar in different alcohol solvents or in water-alcohol mixtures. Water and ethanolic extracts have a large number of health-beneficial properties such as antibacterial, anticancer, antidiabetic, antifungal, antihypercholesterolemic, antihypertensive, antiinflammatory, antioxidant, antiparasitic, antiviral, and immunostimulatory activities. Moreover, due to their antioxidant and immunostimulatory properties, aqueous and ethanolic garlic extracts can be used as an aid in conventional anticancer therapy, but more research in this field is needed. In addition to the obvious culinary uses, garlic oil can also be used as an adjuvant agent in conventional therapies for different skin infections and pathologies due to the large amounts of DADS > allyl-methyl trisulfide > trisulfide and di-2-propenyl > DAS, which have antibacterial and antiinflammatory properties. However, the use of such substances as methanol and/or organic solvents is limited due to their toxicity, especially when consumed directly. Filling the gaps in the knowledge of methods for improvement of the stability of bioactive compounds contained in garlic extracts and the selection of the best modes and forms of supplying thereof to organisms yielding a therapeutic effect may contribute to the higher efficiency of the use of garlic extracts. Future research should focus on the improvement of the stability of bioactive compounds obtained from the extracts.

## Figures and Tables

**Table 1:** Summary of methods used in extraction of active components from garlic (*Allium sativum* L.). NDA—no data available.

Type of Extract	Extraction Method	Analyses Performed	References
Aqueous	Distilling the garlic extract solution under reduced pressure	Assessment of antiproliferative properties of copper-enriched garlic extract	[29]
	Pressing extraction	Identification of allicin with anticancer activity	[30]
Methanol	Maceration	Analysis of viability and apoptosis in leukemia cells	[31]
Ethanol	Solvent extraction	Analysis of motor coordination and Purkinje cell count in rats	[32]
	Solvent extraction	Analysis of antibacterial properties against Staphylococcus aureus	[33]
Chloroform	Solvent extraction under reduced pressure	Assessment of the antiinflammatory properties of aged black garlic	[34]
Fresh material	Blended in water	Analysis of NO and interferon-α (IFN-α) levels in plasma	[35]
Freeze-dried material	NDA	Preservation of minced meat	[36]
Oil	Steam distillation	Preservation of minced meat	[36]
	NDA	Analysis of the mechanism of cytotoxicity of DATS in leukemic cells	[37]

**Table 2:** A comparative summary of bioactive ingredients in water and alcohol extracts. Abbreviations: AC—allicin; AS—allyl sulfide; CAE—caffeic acid equivalent; DADS—diallyl disulfide; dw—dry weight; GAE—gallic acid equivalents; GH—garlic shell; MMTS—methanethiosulfonic acid S-methyl ester; NDA—no data available; QUE—quercetin equivalent; TS—thiosulfinates.

Compunds	Solvent	Contents	Extraction Method	Geographic Region	References
AC	Water extracts	42.74 and 50.79 μg/mL	Pressing extraction	Serbia	[9]
	Alcohol extracts	4.39 to 4.56 μg/mL (ethanol)	Pressing extraction	Serbia	
	Alcohol extracts	7068 ppm (ethanol)	Soxhlet extractions	Spain	[55]
MMTS	Water extracts	0.09–0.33 μg/mL	Pressing extraction	Serbia	[9]
	Alcohol extracts	0.45–0.67 μg/mL (ethanol)	Pressing extraction	Serbia	
AS	Water extracts	1.91–4.72 μg/mL	Pressing extraction	Serbia	[9]
	Alcohol extracts	0.21–0.70 μg/mL (ethanol)	Pressing extraction	Serbia	
DADS	Water extracts	0.01–0.03 μg/mL	Pressing extraction	Serbia	[9]
	Alcohol extracts	0.03–0.04 μg/mL (ethanol)	Pressing extraction	Serbia	
TS	Water extracts	6.42 µmol/g (dw)	Uultrasound-assisted extraction	Brazil	[15]
	Alcohol extracts	1.40 µmol/g dw (ethanol)	Ultrasound-assisted extraction	Brazil	
Phenols	Water extracts	3.82 mg/g dw	Ultrasound-assisted extraction	Brazil	[15]
	Alcohol extracts	0.84 GAE/g dw (ethanol)	Ultrasound-assisted extraction	Brazil	
	Water extracts	2.97 mg GAEs/g	Solvent extraction	NAD	[50]
	Alcohol extracts	13 mg GAE/g (50% ethanol); 11.80 mg GAE/g (ethanol); 25 mg GAE/g (50% methanol); 22.83 mg GAE/g (methanol)	Solvent extraction	NAD	
	Water extracts	201.99 to 365.52 μg/g	Blended in water	Spanish	[20]
		231.66 μg/g	Blended in water	Uzbek	
		185.26 μg/g	Blended in water	Thai	
		253.58 μg/g	Blended in water	Burmese	
		201.04 μg/g	Blended in water	Portuguese	
		394.10 μg/g	Blended in water	Chinese	
		223.74 μg/g	Blended in water	Polish	
	Water extracts	0.112 to 0.311 mg/g	Boiling and ultrasound	Iran	[45]
	Alcohol extracts	0.216 to 0.269 mg/g (ethanol)	Boiling and ultrasound	Iran	
	Water extracts	0.285 mg/mL	Blended in water	Nigeria	[53]
	Alcohol extracts	24.81 mg GAE/g (ethanol); 29.72 mg GAE/g (methanol)	Solvent extraction	Bangladesh	[56]
Flavonoids	Water extracts	28.74 mg QUE/mL	Blended in water	Nigeria	[53]
	Alcohol extracts	22.51 mg CAE/g (ethanol); 20.18 mg CAE/g (methanol)	Solvent extraction	Bangladesh	[56]
	Water extracts	0.045 mg QUE/g dry GH	Solvent extraction	NAD	[50]
	Alcohol extracts	0.51 mg QUE/g (50% ethanol); 0.486 mg QUE/g (ethanol); 0.617 mg QUE/g (50% methanol); 0.602 mg QUE/g (methanol)	Solvent extraction	NAD	
Flavonols	Alcohol extracts	12.92 mg QUE/g (ethanol); 11.92 mg QUE/g (methanol)	Solvent extraction	Bangladesh	[56]
Proanthocyanidins	Alcohol extracts	5.13 mg CAE/g (ethanol); 5.17 mg CAE/g (methanol)	Solvent extraction	Bangladesh	[56]

**Table 3:** Biological properties of garlic (*Allium sativum* L.) in certain models.

Type of Action	Model	References
Antibacterial	In vitro	[51,67,68,69,70]
Anticancer	In vitro/In vivo	[6,71,72,73,74,75,76,77,78,79,80]
Antidiabetic	In vivo	[81,82]
Antifungal	In vitro	[25,83,84,85,86]
Antihypercholesterolemic	In vivo/In vitro	[87,88,89]
Antihypertensive	In vivo/In vitro	[90]
Antiinflammatory	In vivo/In vitro	[34,91,92,93]
Antioxidant	In vitro	[68,80,94,95,96,97,98,99,100,101]
Antiparasitic	In vivo/In vitro	[102,103]
Antiviral	In vitro	[104,105,106,107,108]
Hepatoprotective	In vivo	[98,109,110]
Immunostimulatory	In vitro/In vivo	[111,112,113]
Insecticidal	In vivo	[51,114]
Neuroprotective	In vitro	[115,116]

**Table 4:** Anticancer properties of garlic extracts in in vitro studies.

Type of Cancer	Type of Extract	Proposed Mechanism of Action	References
HT29 (human colon adenocarcinoma)	Ethanol	Apoptosis	[136]
CACO-2 (human colon carcinoma)	Aqueous, methanol, ethanol	ROS	[130]
	Crude garlic	Inhibition of cell proliferation	[137]
colo 205 (human colon adenocarcinoma)	Crude garlic	Reduction in cell viability, induction of apoptosis	[138]
32Dp210 (murine myeloid leukemia)	Aqueous	Oxidant stress	[134]
HL-60 (human leukemia)	Aqueous	Cytotoxic effect, apoptosis	[139]
ALL (precursor-B acute lymphoblastic leukemia)	Fresh garlic	Selective cells apoptosis	[140]
U937 (histiocytic lymphoma)	Oil	ROS, apoptotic	[37]
TIB (monocyte/macrophage cell line)	Crude garlic	Inhibition of cell proliferation	[137]
HepG2 (human hepatoma)	Aqueous	Antiproliferative effect, overexpression of p53 and p21 (break of DNA strand)	[29]
	Crude garlic	Inhibition of cell proliferation	[137]
	Heat-aging	Inhibition of cell proliferation	[7]
SGC-7901 (human gastric cancer)	Aged black garlic	Inhibition of cell growth through apoptosis, inhibition of tumor growth in rats, which may result from antioxidant and immunomodulating effects	[141]
AsPC-1 (pancreatic beta cells)	Oil	Pro-apoptotic effect as a result of programmed cell death, cell cycle arrest	[142]
Squamous cell carcinomas (SCC)	Aqueous	Modulating lipid peroxidation, increase in the levels of GSH, GPx, and GST	[143]
U2OS (human bone osteosarcoma epithelial cells)	Ethanol	Reduced proliferation mediated by increased endoplasmic reticulum (ER) stress	[76]
U937 (human histiocytic lymphoma cell line)	Heat-aging	Inhibition of cell proliferation	[7]
Mia PaCa-2 (epithelial cell line)	Oil	Inhibition of cell proliferation	[142]
Sk-mel3 (human melanoma)	Aqueous	Decrease in cell viability	[144]
MCF-7 (human breast cancer)	Aqueous	Decrease in cell viability	[145]
	Fresh garlic	Inhibition of cell growth, change in cell morphology	[72]
	Crude garlic	Inhibition of cell proliferation	[137]
PC-3 (human prostate cancer)	Crude garlic	Inhibition of cell proliferation, cell cycle arrest	[137]
PANC-1 (human pancreatic cancer)	Oil	Inhibition of cell proliferation	[142]
DU145 (human prostate cancer)	Ethanol	Reduced proliferation mediated by increased endoplasmic reticulum (ER) stress	[76]
67NR (cellosaurus cell line)	Ethanol	Reduced proliferation mediated by increased endoplasmic reticulum (ER) stress	[76]
